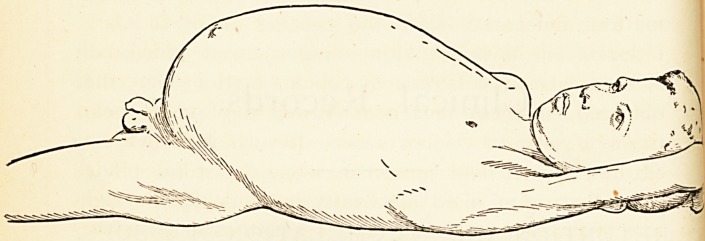# Retention and Accumulation of Fæces

**Published:** 1885-03

**Authors:** J. E. Shaw


### Clinical Records.

---

### RETENTION AND ACCUMULATION OF FÆCES.

By J. E. Shaw, M.B.

J. R., æt. 15½ years, was admitted to the Bristol Infirmary on October 16th, 1884, for swelling of the abdomen attended with pain.

He was stated to have suffered from great constipation and a more or less distended belly from the time of his birth, and had been in the habit recently of having his bowels moved about once a month. There had been an action of the bowels two days before admission to the Infirmary, previous to which there had been no relief for five weeks. On account of the increasing distress and general malaise, he had been obliged to desist for the previous seven weeks from his occupation as a tin-plate worker. The patient was an emaciated, cachectic, ill-developed lad, measuring only 4 ft. 6 in. in height, but of considerable intelligence. He complained of a good deal of pain and discomfort in his abdomen, but was able to take his food well, and had no vomiting or other signs of intestinal obstruction.

On examining his abdomen it was found to be enormously distended *(see outline sketch from photograph taken*

*the day after admission*), the portion of the walls between the epigastrium and umbilicus having been more stretched in proportion than that between the umbilicus and pubes. The circumference of the abdomen was $42\frac{1}{2}$ inches, measured five inches above the umbilicus. Round the lateral and superior regions of the abdomen ran a raised ridge, four or five inches broad, while the central region above the umbilicus was a flat, rather shallow depression, bounded by the aforesaid ridge, as if a ship's cable had been placed beneath the integuments of the periphery of the abdomen. The belly was moderately resonant upon percussion all over, more so in the central depressed area, and palpation of the raised parts gave one very much the idea of a bag containing small potatoes. The liver was pushed up under the ribs and diaphragm, which divided the widely-dilated short thorax from the abdominal cavity; the other abdominal organs presented nothing peculiar to external examination. On exploring his rectum it was found to be empty, and widely dilated; while the lower end of the sigmoid flexure full of hard fæces, was pushed down into the rectum in the manner of an intussusception.

The symptoms not being urgent, he was placed upon moderate doses of aloes and nux vomica, taken twice daily; in the course of three days his bowels began to act slightly, large pieces of dark-coloured fæces being passed. Two days later, in order to facilitate the escape of fæcal matters and to stimulate the contraction of the large intestine, enemata containing oil of rue were added daily to the treatment. Extremely copious motions were then obtained, three or four pounds weight of fæces being passed per diem for a day or two, after which the left side of the abdomen had sunken in, while the still distended ascending colon stood up a lofty hummock in the right side of his belly. As this rapid evacuation of the bowels led to considerable exhaustion, the enemata were discontinued for some days; but returning constipation made them necessary again, with the further addition of a dose of house-mixture every morning a few days later. On Nov. 5th he was seized with severe abdominal pain of a colicky nature, with vomiting and a pinched look in his face, leading one to fear that a block might have occurred in his cæcum; the temperature was not raised, and his bowels did not cease to act, though his aperients were discontinued. On the 6th he was placed upon frequent doses of belladonna, upon which he improved, and on the 9th had a dose of ol. ricini, which was repeated each evening. On the 12th, the simple enema was replaced by one with turpentine, upon which his bowels were moved eight times. From this time no further trouble was met with in obtaining the continued free use of his bowels; but though his large intestine was thus emptied of its solid contents, it was found that the relaxed abdominal walls readily permitted of its dilatation by gaseous accumulation. On Nov. 15th the descending colon measured transversely 5½ inches. He was therefore ordered to have the current from 15 cells of the constantcurrent battery applied across the abdomen every evening. His weight on Nov. 21st was 4 st. 3 ℔.

After this the treatment of his case consisted simply in the determined administration of purgatives, with strychnia, the battery, and enemata, as there was a tendency for fæcal matter to re-accumulate, imparting a doughy feel to his abdomen on palpation, if the energy of the treatment was relaxed. On Dec. 4th, for example, he took in the early morning a draught of mag. sulph. and senna, three doses of an aperient chalybeate mixture with strychnia; in the afternoon he had a simple enema, and at night a pill composed of podophyllin gr. $\frac{1}{4}$ and pil. scammonii co. gr. iv., as well as the battery. Notwithstanding this purgation he rapidly improved in health and strength, and, having now a good appetite, laid on flesh, so that when discharged on Dec. 29th he weighed 5 st. 6℔. His abdomen was reduced to nearly a normal degree of protuberant distension (for more than a month it had been transversely of a normal oval instead of a sinuous shape, the contour of the colon not being visible through the abdominal wall), and had *almost* completely lost the doughy feel. During the last 48 days of his stay in the Bristol Infirmary he had 241 actions of the bowels, or an average of five per diem.

On Feb. 11th, 1885, he exhibited himself at a meeting of the Bristol Medico-Chirurgical Society, greatly improved in health: his abdomen measured 26 inches in circumference above the umbilicus, and was in all respects normal except that at the line of junction with the thorax it was still abnormally wide. He continued to find aperients necessary, and was taking nightly a pill containing podophyllin gr. $\frac{1}{2}$, pil. col. et. hyos. gr. iv.

*Remarks.*—Although so far this case may be considered highly satisfactory, still the boy's life will be for many years in constant jeopardy. The tendency for his large intestine to become a mere cesspool, from which only an occasional overflow takes place, will remain, and his life may terminate either by obstruction, by passage of fæces into his vermiform appendix, or by twisting of the cæcum upon itself. The boy and his friends were warned of the potential danger in which he lives, and urged to be upon their guard against allowing constipation to occur.

Probably he would only be cured permanently by the establishment of an artificial anus communicating with the lower end of his ileum for a period of some months, thereby permitting his colon to lie empty and gradually contract in calibre. In the face of immediate success it seems difficult to insist upon this extreme measure of radical cure just now.

## Figures and Tables

**Figure f1:**